# A T-Type Capacitive Sensor Capable of Measuring 5-DOF Error Motions of Precision Spindles

**DOI:** 10.3390/s17091975

**Published:** 2017-08-28

**Authors:** Kui Xiang, Wen Wang, Rongbo Qiu, Deqing Mei, Zichen Chen

**Affiliations:** 1 Key Laboratory of Advanced Manufacturing Technology of Zhejiang Province, College of Mechanical Engineering, Zhejiang University, Hangzhou 310027, China; 11125034@zju.edu.cn (K.X.); medqmei@zju.edu.cn (D.M.); chenzc@zju.edu.cn (Z.C.); 2 School of Mechanical Engineering, Hangzhou Dianzi University; Hangzhou 310018, China; 161010017@hdu.edu.cn

**Keywords:** precision spindle, error motion measurement, capacitive sensor, integrated structure

## Abstract

The precision spindle is a core component of high-precision machine tools, and the accurate measurement of its error motions is important for improving its rotation accuracy as well as the work performance of the machine. This paper presents a T-type capacitive sensor (T-type CS) with an integrated structure. The proposed sensor can measure the 5-degree-of-freedom (5-DOF) error motions of a spindle in-situ and simultaneously by integrating electrode groups in the cylindrical bore of the stator and the outer end face of its flange, respectively. Simulation analysis and experimental results show that the sensing electrode groups with differential measurement configuration have near-linear output for the different types of rotor displacements. What’s more, the additional capacitance generated by fringe effects has been reduced about 90% with the sensing electrode groups fabricated based on flexible printed circuit board (FPCB) and related processing technologies. The improved signal processing circuit has also been increased one times in the measuring performance and makes the measured differential output capacitance up to 93% of the theoretical values.

## 1. Introduction

Spindles, as core components of high-precision CNC machine tools, provide accurate, repeatable rotary motion [1]. An ideal spindle allows the rotation only in a single degree of freedom, but in actual application, the spindle rotation axis cannot coincide with the theoretical axis due to the machining or assembly errors of the spindle itself and its matching components. Therefore, the spindle not only has a single DOF rotation (assumed to be around the axis *z*), but also has 3-DOF linear translation (*δ_x_*, *δ_y_*, *δ_z_*) and 2-DOF tilt error motions (*ε_x_*, *ε_y_*) [2]. The accurate measurement of the 5-DOF error motions can realize the quantitative assessment of the spindle rotation accuracy. More importantly, it is of great significance in improving the spindle rotation accuracy as well as the performance of the machine tools.

Some optical measurement methods were designed to achieve the simultaneous on-axis measurement of the multi-DOF error motions for the spindle, including laser interferometry or multiple sets of optical subsystems which occupy a large space [3,4,5,6]. A non-contact, high resolution capacitive sensor with a simple probe was also used to build a measurement system [2,7,8,9,10], which has a lower complexity and a better environmental stability compared to the optical methods. However, the probe-type capacitive sensor is one-dimensional measuring probe, so for detecting the multi-DOF error motions by means of a precision ball-bar (reference artifact) installed in the spindle, multiple probes must be configured and mounted. This method still has some problems. Firstly, the probe alignment is a difficult task. Secondly, the sensor outputs are sensitive to the roundness errors of the reference artifact [10], and an error separation technique is needed to deal with the measured data, especially when the error motions to be measured are close to the order of magnitude of the artifact accuracy [11]. Thirdly, due to the “plane (probe end face)-curved surface (reference artifact surface)” measurement combination, the initial gap needs to be reduced to ensure the required amount of capacitance changes when the size of artifact obviously decreases. However, the linearity of the sensor output will decrease [12].

Instead of the probe-type capacitive sensor, a cylindrical capacitive sensor (CCS) is proposed to effectively reduce the influence on measurement results caused by the roundness errors of reference artifact and the sensor alignment errors since the CCS has multiple curved cylindrical electrodes and each electrode has a large sensing area [13]. Ahn et al. presented a CCS which is capable of measuring radial displacement of a rotor [14,15]. Kim et al. designed a CCS to measure the spindle motions of milling machine, and the sensor output signals are used to monitor its dynamic cutting force [16]. However, the above CCS are limited in measurement dimensions, and therefore cannot be used to detect 5-DOF error motions of a spindle. There are still some issues that need improvement. The circumferential gap of curved cylindrical electrodes are still large with respect to the effective sensing range of the sensor, and the thickness of electrodes have not yet been adjusted to a thinner one, which makes the additional capacitance caused by fringe effects accounting for a larger proportion in the measurement results, and reduces the linearity of the sensor output [17,18].

This paper presents a T-type CS with an integrated structure. The proposed sensor is used to achieve on-axis measurement of the spindle 5-DOF error motions simultaneously by integrating electrode groups in the cylindrical bore of the stator and the outer end face of its flange (F_S_) respectively. Based on the new sensor design, the reference artifact (i.e., the rotor of the sensor) can be simplified as a two segment stepped shaft that is easy to manufacture. Because of a good deformability, FPCB, which is commonly used in the fabrication of flexible tactile sensors [19] and toroidal coil of eddy current proximity sensors [20] is employed to produce the sensing electrode groups, in order to realize the integrated fabrication and meanwhile reduce the influence of fringe effects. In the paper, the structure of the T-type CS is firstly described. The measurement principle of the designed sensor is then presented by establishing appropriate mathematical models and a theoretical analysis. Finally, finite element simulation and experimental research are carried out to verify the feasibility of the sensor.

## 2. Measurement Principle

### 2.1. The Structure Model of T-Type CS

**Figure 1:**
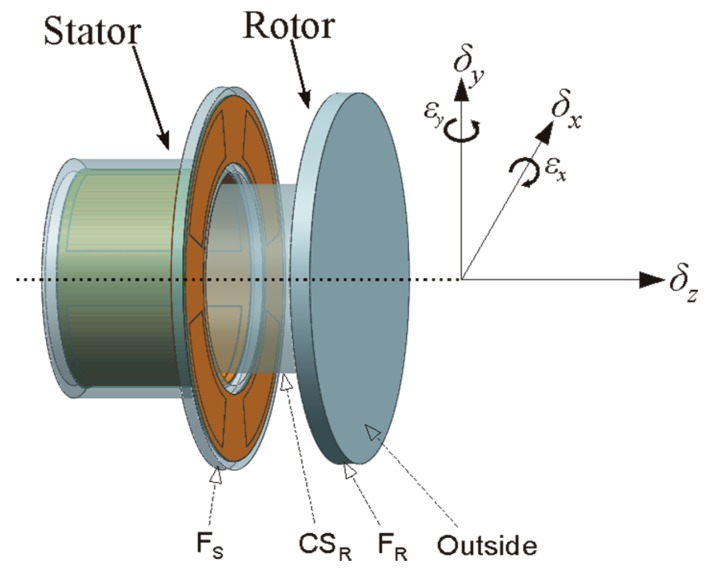
Schematic of T-type CS.

**Figure 2:**
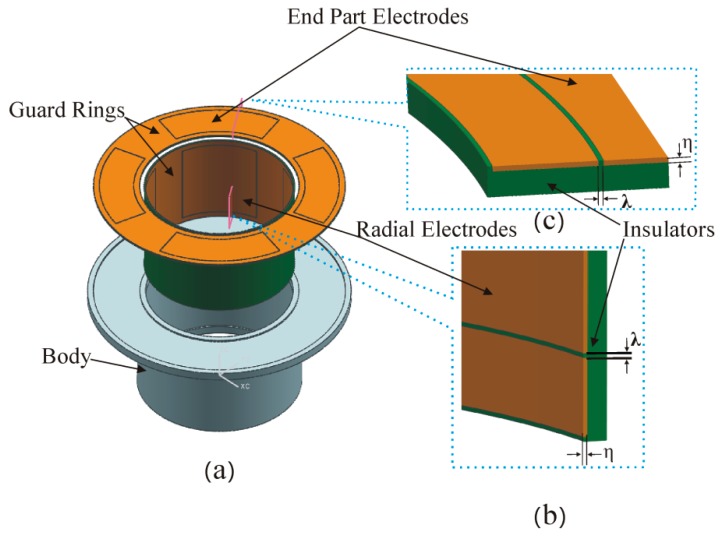
Schematic of the stator: (**a**) The exploded view of the structure; (**b**,**c**) partial cross-sectional view of the stator.

The T-type CS is mainly composed of rotor and stator, and they are coaxially mounted, as shown in Figure 1. The rotor of the sensor acts as a reference artifact to transmit the error motions of the spindle. The rotor is also an excitation electrode (*E_d_*) of the sensor electrically: the cylindrical surface (CS_R_) of the rotor is a radial measuring surface for the detection of radial error motions, and the inside annular end face of the rotor flange (F_R_) is an axial measuring surface for the detection of axial and tilt error motions. As illustrated in Figure 2, the sensing electrode groups include the end part electrode group (EPEG) and the radial electrode group (REG), and they are respectively in the annular groove of the stator end part and in that of stator cylindrical bore. Each group contains four electrodes equally spaced circumferentially which have the same flare angle. The polar angle positions of the end part fan-shaped electrodes are corresponding to that of the radial cylindrical electrodes. Equipotential guard rings are distributed surrounding the periphery of the sensing electrodes with a tiny gap *λ*. They are embedded in the insulators which are taken as intermediate layers and mounted on the stator housing, as shown in Figure 2b,c.

### 2.2. Radial Error Motion Measurement

**Figure 3:**
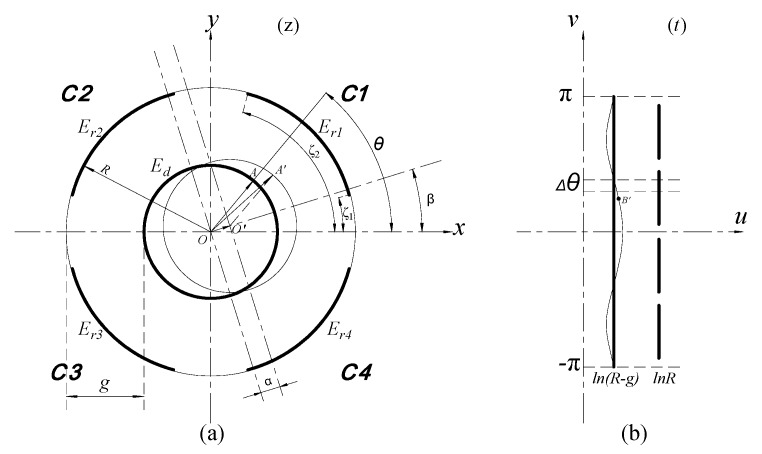
The calculation model of the radial error motions: (**a**) Boundary before transformation; (**b**) boundary after transformation.

The measurement of the spindle radial error motions are actually equivalent to detect the displacements of the rotor along the *x*-axis and *y*-axis of the coordinate plane shown in Figure 3.

This measurement is performed by the REG which is constituted by cylindrical electrodes *E_r_*_1_~*E_r_*_4_. The four cylindrical electrodes with a differential configuration and the radial measuring surface of the rotor make up four capacitors respectively, their corresponding capacitances are C1~C4.

Due to the arc-shaped boundary of the electrode constituting the radial capacitors, the conformal mapping method used in complex function theory is adopted to solve the Poisson Equation in electrostatic fields. Then the arc-shaped boundary which can be expressed as a sets that includes a series of the complex numbers in a complex plane is transformed into the equivalent boundary shown in Figure 3b according to Equation (1) [21,22]; Meanwhile, the initial radial spacing *g* between the rotor and the cylindrical electrodes will also be mapped as an equivalent spacing −ln(1 − *g*/*R*), which could be approximately expressed as *g*/*R* (*g* << *R*):

(1) $$t=\ln z=\ln\left(r\cdot e^{i\theta }\right)=\ln r+\theta \cdot i$$

where *r*, *θ* are respectively the modulus and argument of the complex number corresponding to any point belonging to the boundary, $r=\sqrt{x^{2}+y^{2}}$.

When the rotor has radial displacement with eccentricity *α* at phase angle *β* due to the generated radial run-out of a spindle, the equivalent boundary in the *t* plane that is corresponding to the circular boundary of CS_R_ will be changed to a wavy line shown in Figure 3b, and the point *A′* will also be mapped as the point *B′* on the wavy line. By Equation (1), the approximate expression of the resulting change Δ*u* of the equivalent spacing at point *B′* can be derived as:

(2) $$\Delta u=\frac{\alpha \cos(\theta -\beta )}{R}$$

where *R* is the inside radius of the radial cylindrical electrode.

For the micro-unit that is taken with an equivalent length Δ*θ* at point *B′*, the capacitance of the corresponding parallel plate capacitor is given by:

(3) $$\Delta C_{r}=\frac{\varepsilon_{0}\varepsilon_{r}\Delta \theta }{\left(g-\alpha \cos\left(\theta -\beta \right)\right)/R}$$

where *ε*_0_ is the electric permittivity of vacuum, *ε_r_* is the relative dielectric constant.

Thereby, the differential expression of the output capacitance of a cylindrical electrode with axial width of *w* when the rotor has a radial run-out can be illustrated as:

(4) $$\Delta C_{r}=\varepsilon_{0}\varepsilon_{r}\frac{R\Delta \theta w}{g-\alpha \cos\left(\theta -\beta \right)}$$

Then the output capacitance of the cylindrical electrode in each quadrant is approximately expressed as follows:

(5) $$C1=\varepsilon_{0}\varepsilon_{r}Rw\int_{\zeta_{1}}^{\zeta_{2}}\frac{1}{g-\alpha \cos\left(\theta -\beta \right)}d\theta$$

(6) $$C2=\varepsilon_{0}\varepsilon_{r}Rw\int_{\pi -\zeta_{2}}^{\pi -\zeta_{1}}\frac{1}{g-\alpha \cos\left(\theta -\beta \right)}d\theta$$

(7) $$C3=\varepsilon_{0}\varepsilon_{r}Rw\int_{\pi +\zeta_{1}}^{\pi +\zeta_{2}}\frac{1}{g-\alpha \cos\left(\theta -\beta \right)}d\theta$$

(8) $$C4=\varepsilon_{0}\varepsilon_{r}Rw\int_{2\pi -\zeta_{2}}^{2\pi -\zeta_{1}}\frac{1}{g-\alpha \cos\left(\theta -\beta \right)}d\theta$$

Based on Equations (5)–(8), the expressions of the differential output capacitance of the REG are respectively established as follows:

(9) CrX=C1+C4−C2−C3

(10) CrY=C1+C2−C3−C4

where CrX, CrY are respectively corresponding to the components *δ_x_*, *δ_y_* of the displacement *α* in the *X* and *Y* directions.

Further, the expressions for estimating the radial error motions of the spindle can be derived as follows:

(11) $$\delta_{x}=f_{x}\left(C1+C4-C2-C3\right)$$

(12) $$\delta_{y}=f_{y}\left(C1+C2-C3-C4\right)$$

where *f_x_*, *f_y_* are respectively denoted the transition functions of measured capacitance of the cylindrical electrodes and the radial run-out in the *x*, *y* direction.

### 2.3. Axial Error Motion Measurement

**Figure 4:**
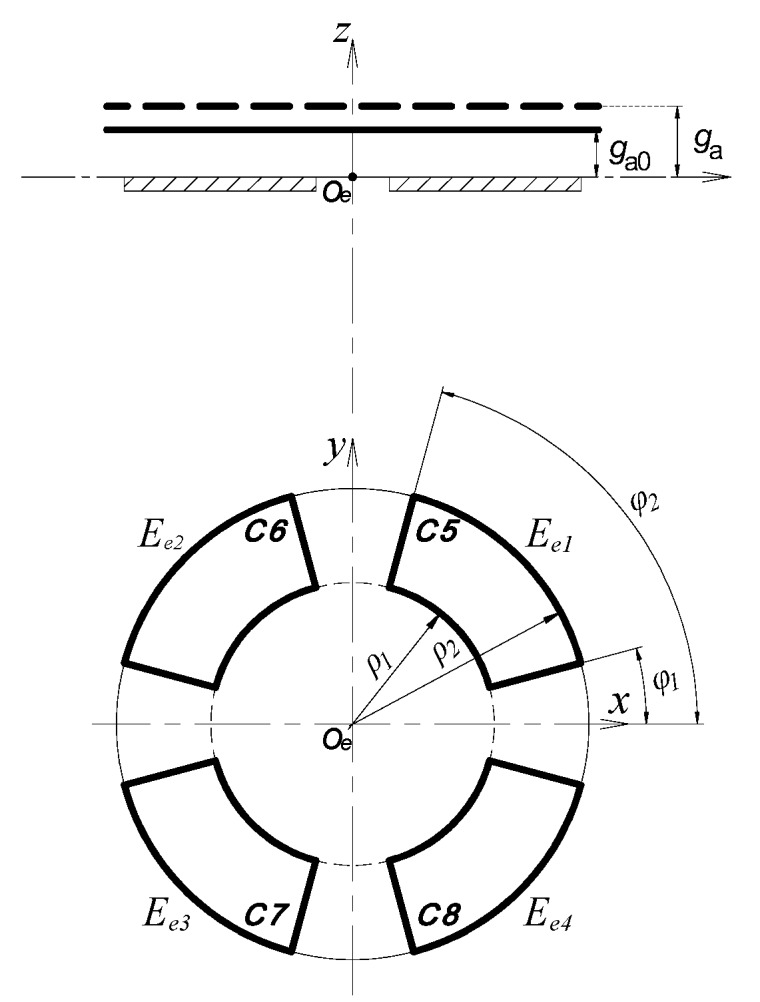
The calculation model of the axial error motion.

As shown in Figure 4, the fan-shaped electrodes (*E_e_*_1_~*E_e_*_4_) of the EPEG are also distributed in the same configuration as radial electrodes. They and the axial measuring surface of the rotor make up four capacitors respectively, the corresponding capacitance are C5~C8. When the rotor has an axial error motion along the *z-*axis, the axial spacing of these capacitors will be changed from the initial value *g_a_*_0_ to *g_a_*.

Thus, the variation quantity of the sum of the output capacitance of the end part electrode is given by:

(13) $$\begin{array}{ll} \Delta \mathrm{CeZ} & =\mathrm{CeZ}\prime -\mathrm{CeZ}\\ & =4\varepsilon_{0}\varepsilon_{r}S_{e}(\frac{1}{g_{a0}+\Delta z}-\frac{1}{g_{a0}}) \end{array}$$

where *S_e_* is the area of the end part fan-shaped electrode, Δ*z* = *g_a_* − *g_a_*_0_; CeZ = C5 + C6 + C7 + C8.

According to Equation (13), the expression for estimating the axial error motion of the spindle can be derived as:

(14) $$\delta_{z}=f_{z}\left(C5+C6+C7+C8\right)$$

### 2.4. Tilt Error Motion Measurement

**Figure 5:**
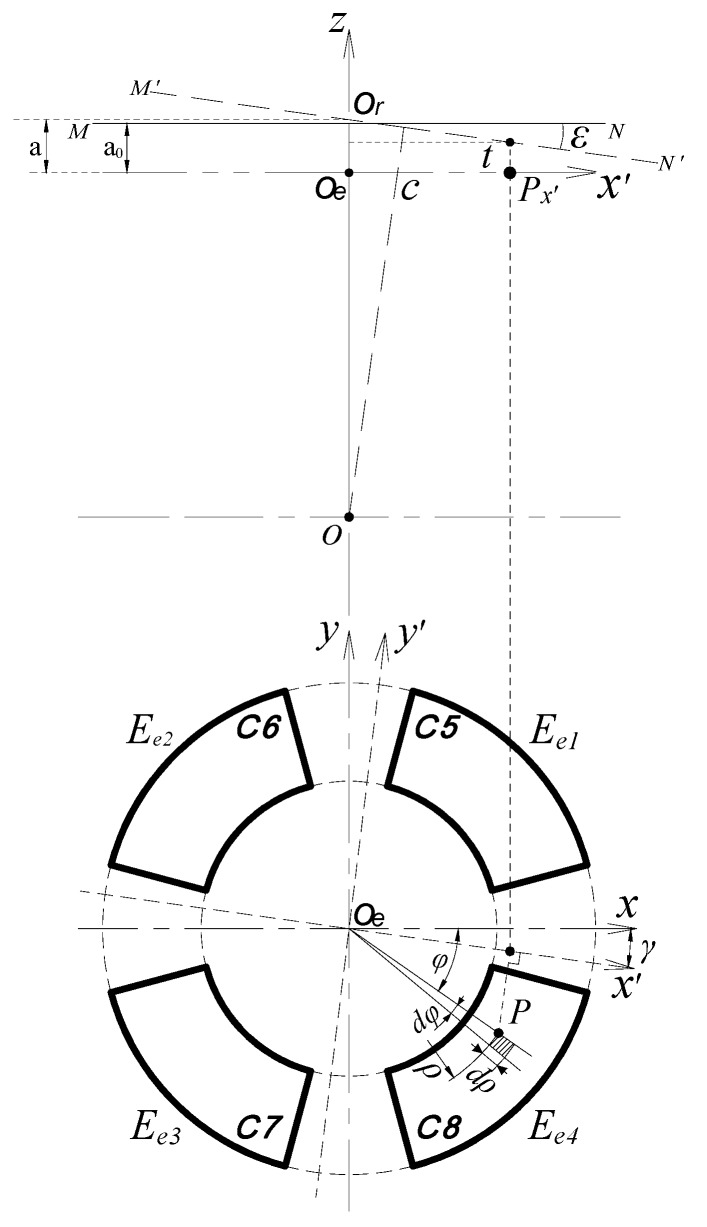
The calculation model of the tilt error motions.

Based on the structural model of T-type CS, the calculation model for the measurement of tilt error motions by EPEG is established in Figure 5. The line segment *MN* is used to represent the axial measuring surface of the rotor (i.e., the inside annular end face of the rotor flange), and its perpendicular bisector is the medial axis of the rotor.

It is assumed that the rotor tilt point is the point *O* in Figure 5 as the spindle produces a tilt error motion. When the rotor has a tilt displacement with amplitude *ε* at the yaw angle *γ* relative to the *z*-axis, the segment *MN* will correspondingly be tilted to the position indicated by segment *M′N′*, and intersects the *z*-axis at point *O_r_*. This process is equivalent to the horizontal plane A located at point *O_r_* produces an equal tilt motion about the *y′*-axis (the angle of it relative to the *y*-axis is *γ*), and meanwhile, because the end part fan-shaped electrodes are always in the projection region of the axial measuring surface (this is guaranteed by their structural parameters), i.e., the effective measuring area of the fan-shaped electrodes are unchanged during the process, thereby the capacitance C5~C8 could be calculated by means of the plane A.

In the boundary region of the fan-shaped electrode, the micro-plane element ΔB_P_ at any point *P* is taken as the solving unit (the area of this micro-plane is denoted as Δ*S_P_*). At point *P*, create a line parallel to the *z*-axis, and the line intersects with a micro-plane ΔA_Q_ at point *Q*. The micro-plane ΔA_Q_ is produced with an opposite region to the micro-plane ΔB_P_ in plane A (the area of it is also denoted as Δ*S_Q_*). With respect to the entire boundary region of the fan-shaped electrode, the micro-planes ΔB_P_ and ΔA_Q_ can be considered as parallel to each other, thus there is $\Delta S_{P}≅\Delta S_{Q}$, and the spacing between them can be substituted by the distance *τ* of points *P* and *Q*.

In the polar coordinate system, the Δ*S_P_* is approximately given by:

(15) $$\Delta S_{P}=\rho d\varphi \cdot d\rho$$

where *ρ* and *ϕ* are respectively denoted the polar radius and polar angle at the point *P*.

As shown in Figure 5, *P_x′_* is the projection point of *P* on the *x′*-axis. At point *P_x′_*, create a line perpendicular to the *xO_e_y* plane, and the line intersects with *M′N′* at point *Q_x′_*. For the points of the line which is located on the plane A and parallel to the *y′*-axis, the distance from them to the fan-shaped electrode are all equal, so the distance *t* between the points *P_x′_* and *Q_x′_* is equivalent to the unsolved spacing *τ* between the micro-planes ΔB_P_ and ΔA_Q_. The expression for calculating the distance *t* is derived as:

(16) t=a−k⋅ρcos(φ−γ)

where *a* is the distance from the intersection point *O_r_* to the *xO_e_y* plane, *k* = tan(*ε*).

According to Equations (15) and (16), the capacitance of the plane-parallel capacitor composed of the micro-planes ΔB_P_ and ΔA_Q_ is:

(17) $$\Delta C_{e}=\frac{\varepsilon_{0}\varepsilon_{r}\Delta S}{t}=\varepsilon_{0}\varepsilon_{r}\frac{\rho d\varphi \cdot d\rho }{a-k\cdot \rho \cos(\varphi -\gamma )}$$

Through the integration of Equation (17) over the area of the fan-shaped electrode, the output capacitance of the fan-shaped electrode *E_e_*_4_ is approximately expressed as:

(18) $$C8=\varepsilon_{0}\varepsilon_{r}\int_{{2\pi -\varphi }_{2}}^{{2\pi -\varphi }_{1}}\int_{\rho_{1}}^{\rho_{2}}\frac{\rho d\rho }{a-k\cdot \rho \cos(\varphi +\gamma )}d\varphi$$

where (*ϕ*_2_ − *ϕ*_1_) are the angular size of fan-shaped electrodes, and *ρ*_1_, *ρ*_2_ are the inside and outside radius of fan-shaped electrodes respectively, as shown in Figure 4.

By analogy with the electrode *E_e_*_4_, the output capacitance of the other electrodes can be approximately expressed as follows:

(19) $$C7=\varepsilon_{0}\varepsilon_{r}\int_{{\pi +\varphi }_{1}}^{{\pi +\varphi }_{2}}\int_{\rho_{1}}^{\rho_{2}}\frac{\rho d\rho }{a-k\rho \cos(\varphi +\gamma )}d\varphi$$

(20) $$C6=\varepsilon_{0}\varepsilon_{r}\int_{{\pi -\varphi }_{2}}^{{\pi -\varphi }_{1}}\int_{\rho_{1}}^{\rho_{2}}\frac{\rho d\rho }{a-k\rho \cos(\varphi +\gamma )}d\varphi$$

(21) $$C5=\varepsilon_{0}\varepsilon_{r}\int_{\varphi_{1}}^{\varphi_{2}}\int_{\rho_{1}}^{\rho_{2}}\frac{\rho d\rho }{a-k\rho \cos(\varphi +\gamma )}d\varphi$$

Summing the Equations (18)–(21), the distance *a* from the intersection point *O_r_* to the *xO_e_y* plane can be derived as:

(22) $$a=\frac{4\varepsilon_{0}\varepsilon_{r}b\cdot (\varphi_{2}-\varphi_{1})}{\mathrm{CeZ}}$$

where, $b=\frac{\rho_{2}^{2}-\rho_{1}^{2}}{2}$.

Based on Equations (18)–(21), the expressions of the differential output capacitance of the EPEG are respectively established as follows:

(23) CeX=C5+C6−C7−C8

(24) CeY=C5+C8−C6−C7

where CeX, CeY are respectively corresponding to the components *ε_x_* (around the *x*-axis), *ε_y_* (around the *y*-axis) of the tilt displacement.

Further, the expressions for estimating the tilt error motions of the spindle can be derived as follows:

(25) $$\varepsilon_{x}=f_{ex}\left(C5+C6-C7-C8\right)$$

(26) $$\varepsilon_{y}=f_{ey}\left(C5+C8-C6-C7\right)$$

where *f_ex_*, *f_ey_* are respectively denoted the transition functions of the measured capacitance of the fan-shaped electrodes and the tilt displacements about the *x*, *y* axis.

### 2.5. Coupling Effect Analysis for the Multi-DOF Error Motions

#### 2.5.1. Effect of Tilt Motion on the Output of Radial Cylindrical Electrode

**Figure 6:**
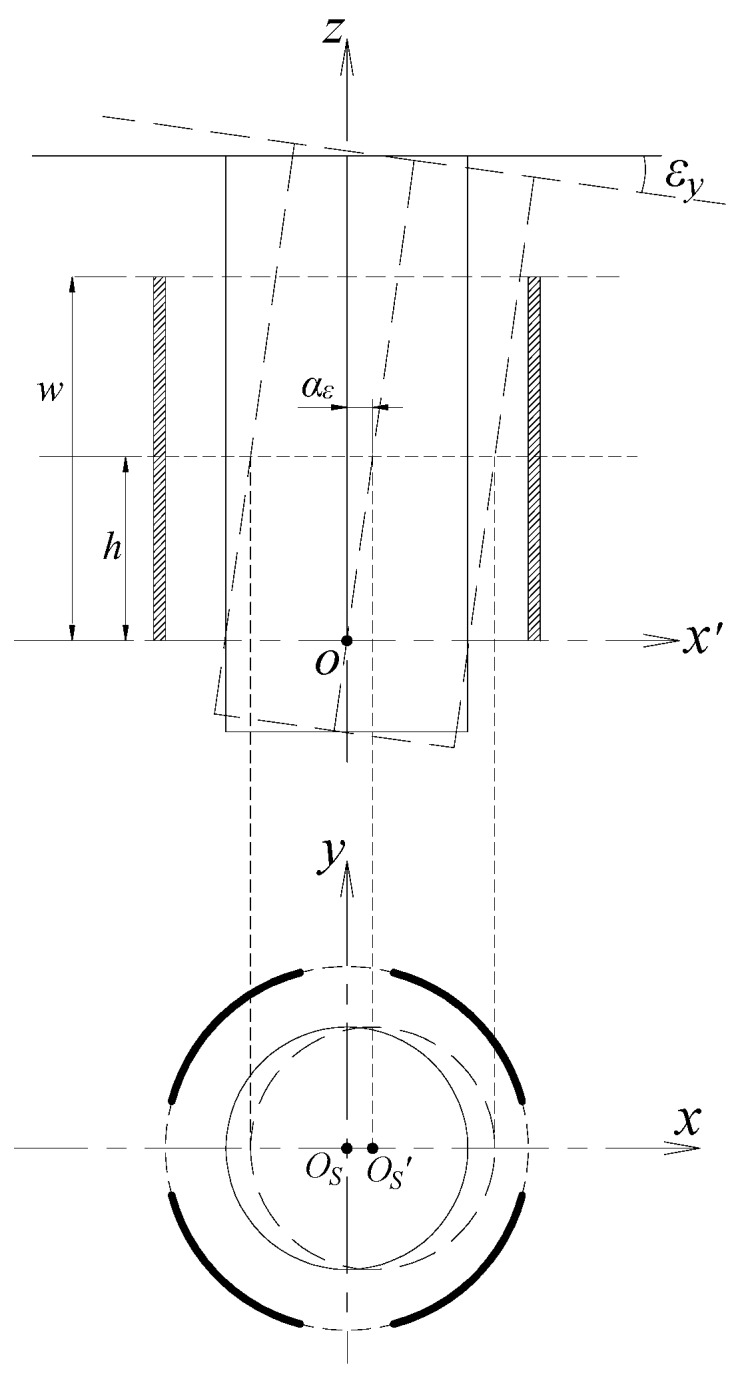
Schematic of the effect of tilt motion on the radial error motion measurement.

It is assumed that the rotor has a tilt displacement *ε_y_* about the *y*-axis, the shape of the horizontal cross-section of the rotor shaft segment (covered by surface CS_R_) which has a height *h* to the bottom of the radial electrode is shown in Figure 6. The shape is an ellipse with the point *O_s_′* as the center, and the length *l_a_* of its major axis is:

(27) $$l_{a}=\frac{R_{cs}}{\cos\varepsilon_{y}}$$

The tilt displacement produced during the rotation of the high precision spindle is very small, thus there is *l_a_* ≈ *R_CS_*, and the ellipse obtained in Figure 6 can be approximated as a circle with radius *R_CS_*, which is equivalent to the circle with radius *R_CS_* located at the center point *O_s_* is shifted to the point *O_s_′*.

Taking into account the effect of tilt *ε_y_*, the approximate expression of the output capacitance of the cylindrical electrode *E_r_*_1_ can be derived as:

(28) $$\begin{array}{c} {C1|}_{\varepsilon_{y}}=\varepsilon_{0}\varepsilon_{r}R\int_{0}^{h}\int_{\zeta_{1}}^{\zeta_{2}}\left[1+h\cdot k_{y}\cos(\theta -\beta )\right]d\theta dh\\ =\varepsilon_{0}\varepsilon_{r}R\int_{\zeta_{1}}^{\zeta_{2}}[h+\frac{h^{2}}{2}k_{y}\cos(\theta -\beta )]d\theta \end{array}$$

where, *k_y_* = tan(*ε_y_*), *h∙k_y_ = α_ε_*.

By analogy with Equation (28), the approximate expression of the output capacitance of the other cylindrical electrodes under the tilt *ε_y_* can be obtained. When the rotor has tilt displacement under different yaw angles (γ*_i_*), the value of the angle *β* in this equation is taken as γ*_i_* correspondingly.

#### 2.5.2. Effect of Tilt Motion on the Measurement of Axial Error Motion

**Figure 7:**
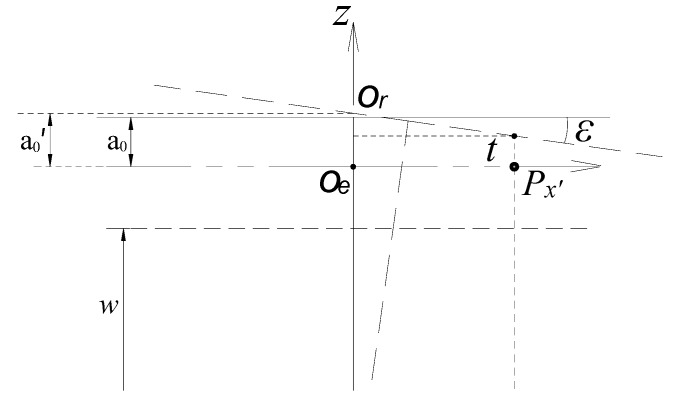
Schematic of the effect of tilt motion on the axial error motion measurement.

As shown in Figure 7, when the rotor has a tilt displacement, its axial measuring surface will intersect the *z*-axis at point *O_r_*. The initial value of *a* will be changed into a_0_’, which makes a nominal change appear for the axial displacement.

Referring to the difference operation method used in calculation of radial displacement, a relatively more accurate equation for the calculation of parameter *a* is given by:

(29) $${a|}_{sub}=\frac{1}{1-\frac{{\mathrm{CeZ}|}_{non}-{\mathrm{CeZ}|}_{esl}}{4}\cdot m}$$

where, $m=\frac{1}{\varepsilon_{0}\varepsilon_{r}b(\varphi_{2}-\varphi_{1})}$, $b=\frac{\rho_{2}^{2}-\rho_{1}^{2}}{2}$; CeZ|*_non_* is the sum of the capacitances of the end part electrodes without rotor tilt and CeZ*|_esl_* is just inversely.

As for the rotor which has a tilt displacement of about 200 arc-secs, the calculated value of the nominal change of the displacement *δ_z_* which is caused by the tilt motion is about 0.02 μm according to the sensor model. The tilt displacement of a high precision spindle is much less than the above value, thus the effect of it on the measurement of axial error motion is very small.

#### 2.5.3. Coupling Effect of Radial and Axial Error Motion under Tilt Displacement

**Figure 8:**
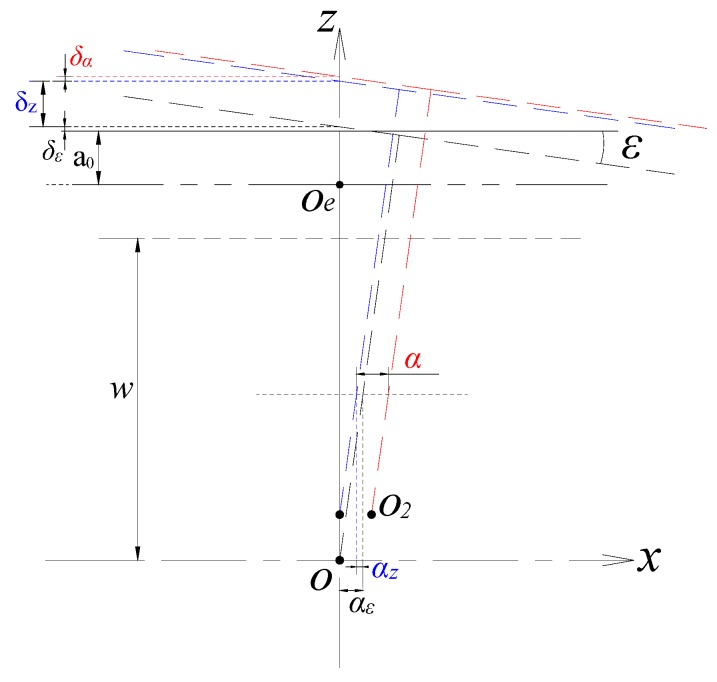
Schematic of the coupling effect of radial and axial error motion.

It is assumed that the position of the rotor is moved from the reference point *O* to the point *O*_2_ due to the multi-DOF error motions (*ε*, *δ_z_* and *α*) of a spindle, as shown in Figure 8. The measurement of tilt error motion is not affected by the radial and axial error motions because the latter are both translational movements.

For the measurement of radial error motion *α*, an additional radial displacement *α_ε_* is generated on the horizontal cross-section due to the tilt displacement of the rotor; meanwhile, as shown in Figure 8, the axial displacement is also accompanied by an additional displacement *α_z_* in the radial displacement direction. The actual measured output capacitance of the radial cylindrical electrode will include the parts caused by the above additional radial displacements. The output capacitance produced by the displacement *α_ε_* can be solved according to Equation (28). For the additional displacement *α_z_*, there is *α_z_* = *δ_z_*∙tan(*ε*). For example, when the rotor has an axial displacement of 200 μm under the tilt displacement with an amplitude 200 arc-secs, the value of *α_z_* is about 0.2 μm. Due to the values of *δ_z_* and *ε* of the high precision spindle are much lower than the above values, the resulting value of *α_z_* is very small, i.e., the effect of the axial displacement shown in Figure 8 on the measurement of radial error motion is quite slight.Thus, after subtracting the output capacitance produced by displacement *α_ε_*, the capacitance value corresponding to the radial displacement *α* can be obtained.

As shown in Figure 8, the additional displacements in the axial direction generated by radial runout and tilt motion are *δ_α_*, *δ_ε_*, respectively. For the displacement *δ_α_*, there is also *δ_α_* = *α*tan(*ε*) and its value is equally quite small; the effect of the latter has been given in the analysis of Figure 7. Thus, the corresponding value of the axial displacement which is obtained by solving Equation (14) can meet the needs for the evaluation of the spindle axial error motion.

## 3. Finite Element Simulation and Analysis

**Figure 9:**
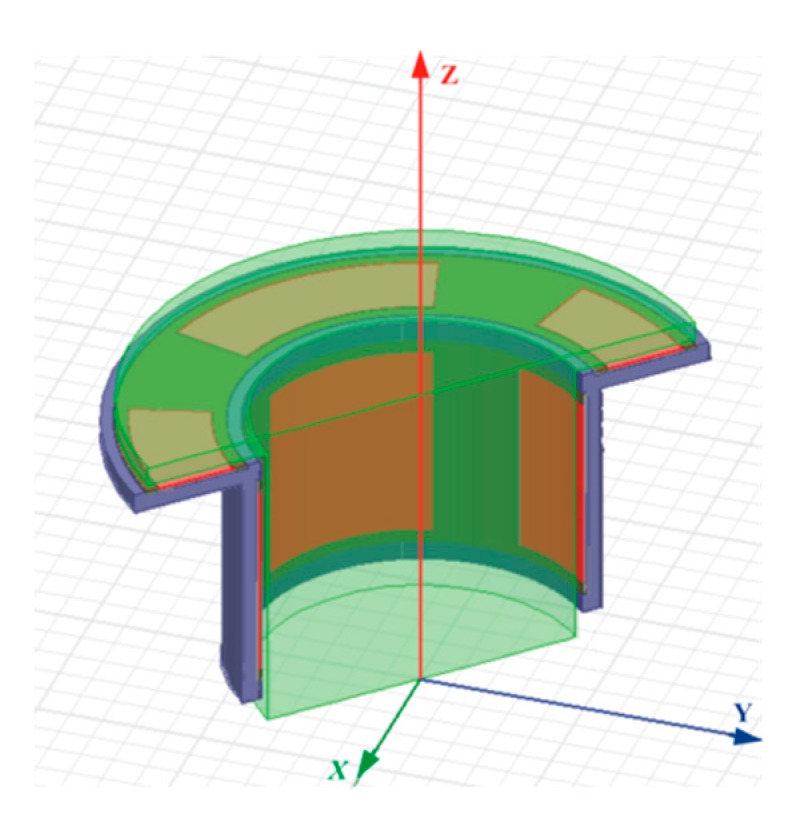
Simulation model of T-type CS.

**Figure 10:**
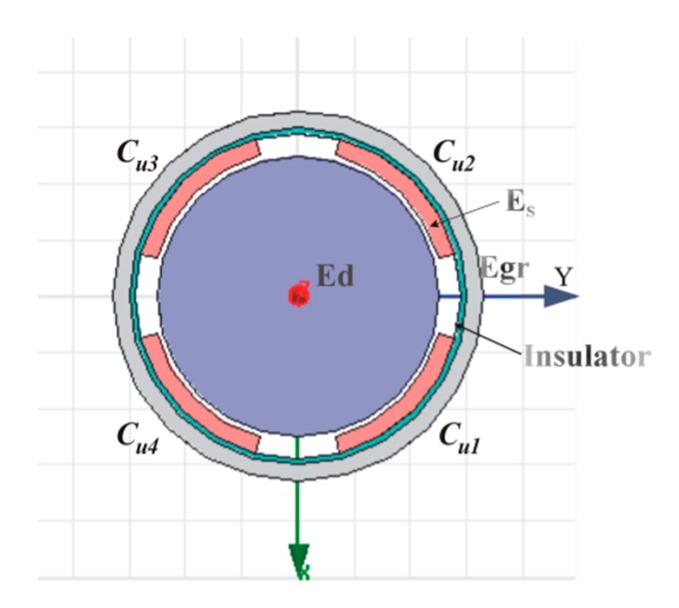
The cross-sectional view of previously developed CCS (2-DOF). The thickness of its sensing electrode *Es* is 3 mm.

In this part, electromagnetic analysis software Maxwell 12 is used for the simulation, to verify the theoretical model. Meanwhile, the effects on the sensor output characteristics when the parameters of its three-dimensional entities such as size or position change are also investigated. The simulation model established according to the parameters listed in Table 1 is shown in Figure 9. Figure 10 shows the main structure of a previously developed CCS capable of detecting 2-DOF error motions, and it is acted as a comparative reference in the following analysis.

When the rotor has an axial error motion, the capacitances of end part capacitors change as the same as the capacitance variation of a parallel plate capacitor appearing a change of spacing. The output characteristics of the parallel plate capacitor are clear. Therefore, the radial error motions and the tilt error motions are mainly investigated through the simulations in this section. Note that C*t is theoretical calculated value and C*f is simulation value in the results.

### 3.1. Radial Error Motions Simulation

Consider the following case that the spindle has a radial runout in y = 0, y = 0.5x and y = x respectively, and study the relationship between the radial cylindrical electrode output capacitance and the displacement of rotor (i.e., excitation electrode, see in Figure 1). In the simulation, *dx* is defined as a component of the spindle radial runout *α* in the *x*-axis direction. The range of the displacement *dx* is set to be (−0.2, 0.2) mm, and the step is 0.01 mm.

As the relevant parameters shown in Table 1, the proposed T-type CS for 5-DOF detection remains the same measuring area of the radial cylindrical electrode as the previously developed CCS and the equal parameters *ζ*, *R* and *w* too. Besides, the initial radial spacing *g* is also identical.

**Figure 11:**
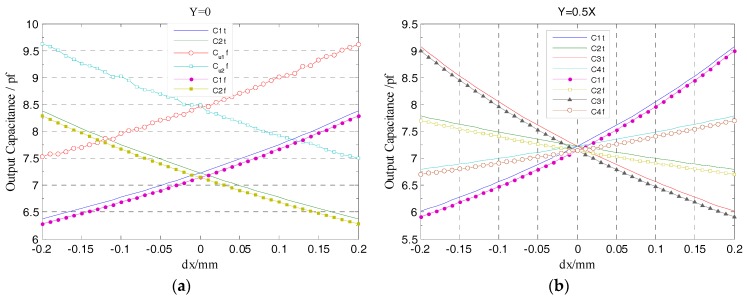
Simulation and theoretical value comparison for the output capacitance of the radial cylindrical electrodes: (**a**) Comparing with the previously developed CCS (2-DOF); (**b**) the comparison of each quadrant cylindrical electrode.

However, the radial cylindrical electrodes of the designed T-type CS are significantly reduced in thickness (*η_r_* = 0.1 mm) relative to the previously developed CCS (3 mm), they are distributed in the co-cylindrical surface with the guard ring (see Figure 2a), and the gap *λ* between these radial electrodes and the guard ring is shortened to 0.1 mm, which effectively improve the shielding effect of the equipotential guard ring and significantly reduce the additional capacitance generated by fringe effects. Thus, as shown in Figure 11a, the simulation output capacitances of radial electrodes vary more close to the theoretical values. Note that C_u1_f, C_u2_f denote the simulation output capacitance of the previously developed CCS.

Figure 11b shows the variation of the output capacitance of radial cylindrical electrodes when the rotor has displacement along y = 0.5x direction. At both sides of the origin (i.e., *dx* = 0), the output capacitance of two cylindrical electrodes in the staggered quadrants (such as C1 and C3) are symmetrically distributed, which is consistent with the symmetrical distribution of the cylindrical electrodes in the coordinate system. Moreover, the simulation output curve of cylindrical electrode in each quadrant show a good agreement with the theoretical value.

**Figure 12:**
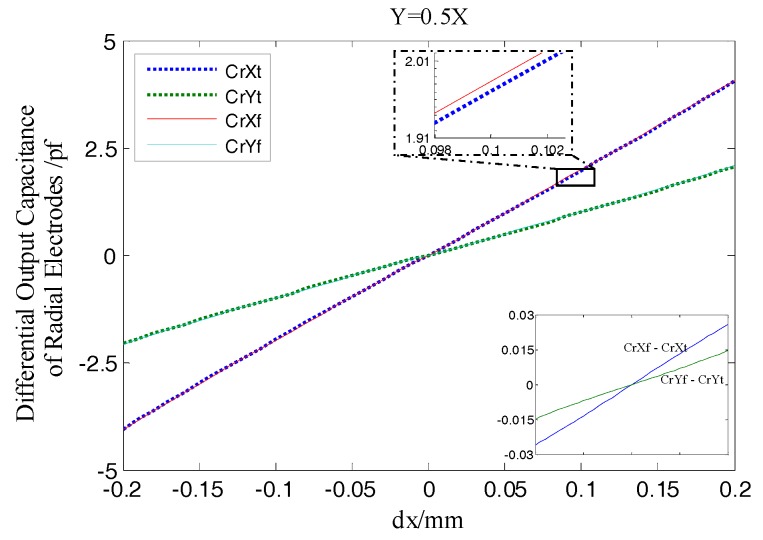
Simulation and theoretical results comparison of radial differential output capacitance.

As shown in Figure 12, the differential output capacitance of the REG in the *X*, *Y* direction change linearly as the rotor has displacement, which is different from the non-linear variation of the output capacitance of a single cylindrical electrode. Moreover, there is no relative offset between the simulation curve and the theoretical one. Furthermore, the additional capacitance due to fringe effects changes with the radial spacing variation, which results in the simulation value and theoretical one in this figure has a difference approximately 0.02 pf when the rotor at the maximum displacement. Compared to the change in radial differential output capacitance per unit displacement (it is about 0.2 pf), the difference has little impact on the measurement results. The simulation results show that, using differential configuration is helpful to eliminate the original additional capacitance of a single electrode at the origin which is caused by the fringe effects, and the measurement accuracy and linearity of the sensor are effectively improved.

**Figure 13:**
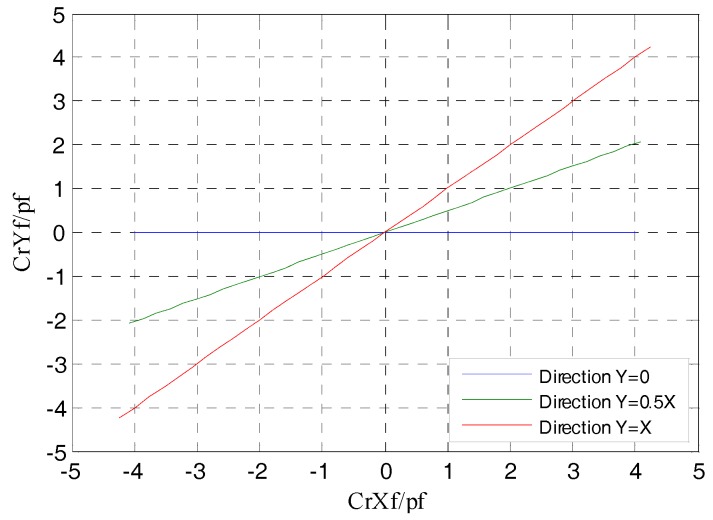
CrYf/CrXf in the different radial runout directions.

From Figure 13 it can be observed that, the relative change ratio of CrYf to CrXf is consistent with the rotor displacement direction in simulation. That is, the differential output capacitance of the REG in the *X* and *Y* direction can accurately reflect the displacement of the rotor in the corresponding direction, so as to realize the effective measurement of the spindle radial error motions.

### 3.2. Tilt Error Motions Simulation

**Figure 14:**
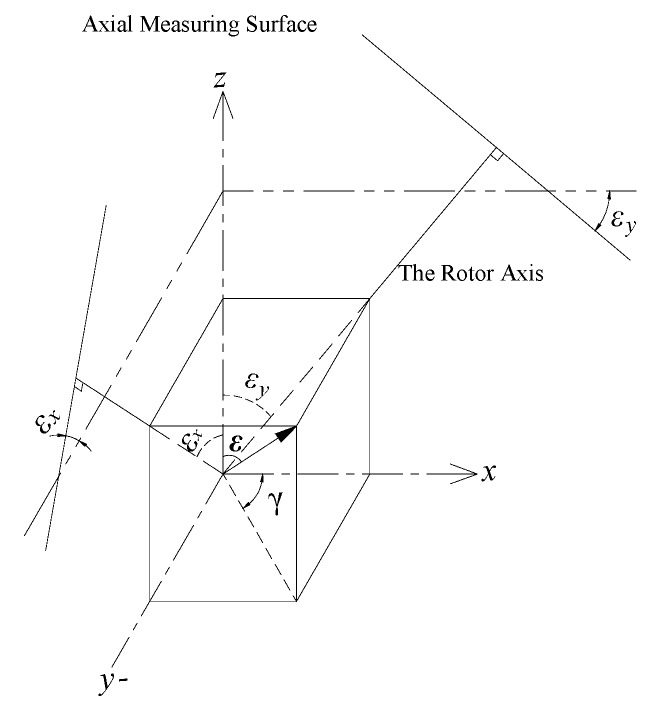
Schematic diagram of tilt error motion decomposition.

In order to simplify the parameter settings in simulation, the amplitude *ε* and yaw angle *γ* in the calculation model are taken as the main parameters to analyze the relationship between the output capacitance of the end part fan-shaped electrode and the tilt displacement of the rotor. According to the geometric model in Figure 14, the tilt *ε_x_*, *ε_y_* of the spindle can be respectively obtained from the decomposition of *ε* in the subsequent calculations.

In the simulation, the variation range of the amplitude *ε* is set to be (−15, 15) arc-min, and the step is 1 arc-min; the yaw angle *γ* is taken as γ_1_ = 15, γ_2_ = 30 and γ_3_ = 45 degree for comparative analysis, which represents the angle between the preset yaw direction and the *x*-axis. The initial gap between the axial measuring surface of the rotor and the end part electrodes is 1 mm, which is equivalent to the initial radial spacing *g* in radial error motions simulation. The tilt point of rotor is set to consistent with the theoretical modeling, that is, the point *O* in Figure 5.

**Figure 15:**
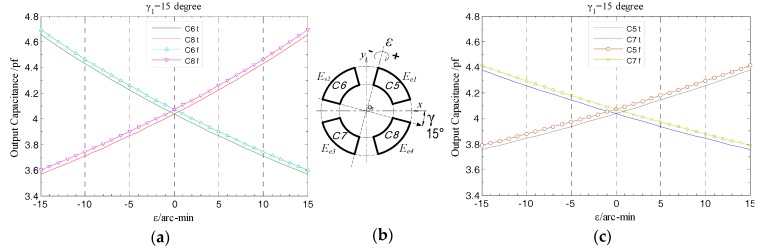
Simulation and theoretical results comparison for the output capacitance of the end part fan-shaped electrodes: (**a**) The 2nd, 4th quadrant; (**b**) tilt schematic; (**c**) the 1st, 3rd quadrant.

When the rotor has a tilt displacement with amplitude *ε* relative to the *z*-axis at yaw angle γ_1_, as shown in Figure 15b, there are part of the micro plane elements ΔB*_i_* (*i* = 1, 2, .., k) located in the boundary region of the electrode in the 2nd or 4th quadrant, whose distance to the tilt axis is larger than that similar elements in the 1st or 3rd quadrant. According to Equation (16), the gap between the former and the axial measuring surface of the rotor changes larger than the latter under the same tilt displacement. As a result, the output capacitance of the electrode *E_e_*_2_ or *E_e_*_4_ generates a relatively large variation over the entire tilt displacement range (see Figure 15a,c). In view of the results, as for the output capacitance of the electrode in each quadrant, the changing trend of the simulation value and that of the theoretical one has well consistency; similarly, the output capacitance curves of the two end part electrodes in the staggered quadrants are symmetrically distributed on both side of the reference position (*ε* = 0 arc-min).

**Figure 16:**
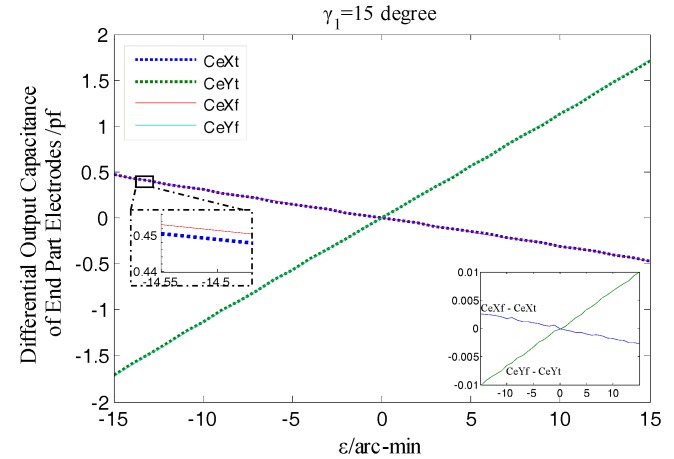
Simulation and theoretical results comparison of end part differential output capacitance.

As shown in Figure 16, the differential output capacitance of the EPEG is also linearly changed due to the tilt displacements of the rotor around the *x* and *y* axis (i.e., the component of *ε*: *ε_x_*, *ε_y_*), and there is no relative offset between the simulated values and the theoretical curve, which indicates that the differential configuration is also effective for the EPEG to measure the tilt displacements. What’s more, the tilt displacement measurement here is essentially a measurement of spacing variation, thus the additional capacitance caused by fringe effects at the maximum tilt displacement has a slight change compared to the initial value without tilt, which results in that the simulation output value at this position is slightly larger than the theoretical value (about 0.01 pf).

According to Equations (23) and (24), the yaw angle of the rotor can be derived as:

(30) $$\tan\gamma =-\frac{\mathrm{CeX}}{\mathrm{CeY}}$$

**Figure 17:**
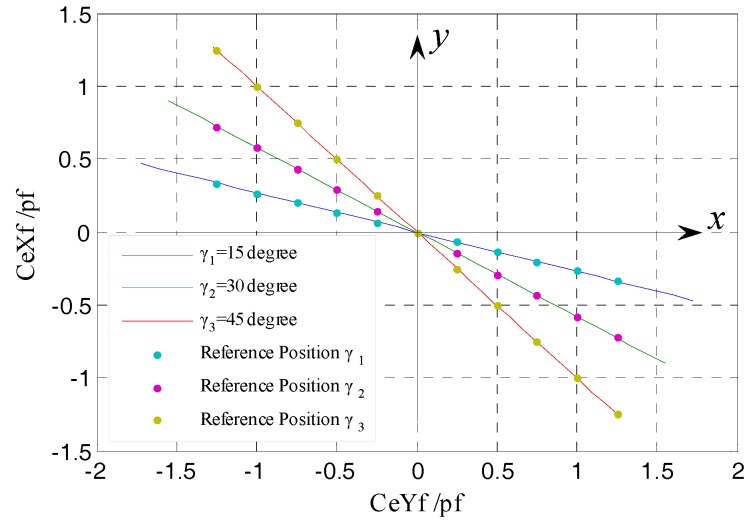
CeXf/CeYf under the different yaw angles.

Based on the line which is plotted by taking CeX, CeY as dependent variable and variable respectively, the yaw direction can be obtained. It can embody the degree of coincidence between the values of yaw angle belonging to a set of tilt displacement and the given yaw angle γ*_i_* (*i* = 1, 2, 3), from a global point of view. As illustrated in Figure 17, the yaw direction obained in simulation is consistent with the preset yaw direction (see reference point), i.e., the simulation values of yaw angle belonging to a set of tilt displacement are in line with expectation. Referring to Figure 14, this means that the differential output capacitance CeXf, CeYf can accurately reflect the tilt *ε_x_*, *ε_y_* of the rotor, thus it is feasible to realize the effective measurement of the spindle tilt error motions by utilizing the EPEG.

According to the above analysis, the predicted output of T-type CS obtained by the proposed mathematical model is in good agreement with simulation results. Both of them will provide the guidance for the design and fabrication of the sensor.

## 4. Experimental Validation

The rotary motion of the working spindle can actually be seen as a combination of multi-DOF displacements appearing on a series of successive time points. During the sensor prototype design stage, in order to facilitate the verification and analysis of its measurement effect, the experiment scheme we adopted is that the continuous rotary motion of the rotor is decomposed into quasi-static motions in different degrees of freedom and then measured respectively. The comprehensive evaluation of the T-type CS will be carried out according to the measurement results subsequently.

**Figure 18:**
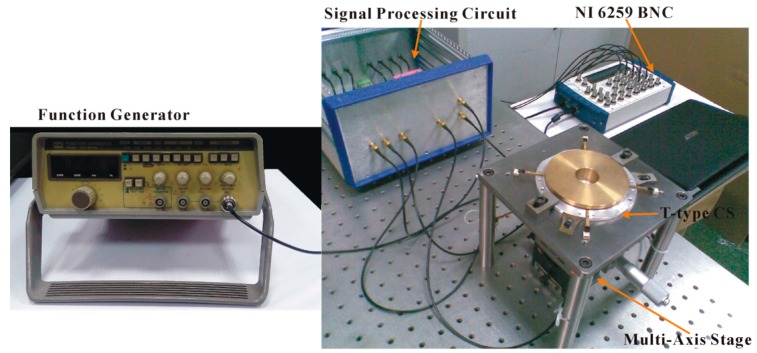
Experimental test system.

The composition of the entire experimental system is shown in Figure 18. The 10-kHz sine wave produced by the function generator GFG-8016G is used as the excitation signal of the capacitive sensor. The stator comprising sensing electrodes is coaxially mounted on the upper support plate relative to an auxiliary support part for the installation of the rotor. The centering between the stator and the auxiliary support part was achieved by inserting a positioning part of tubular cylindrical shape between them. The finished outer reference cylindrical surface of this positioning part has a high coaxiality with its inner bore. After the positioning part is removed, the rotor is coaxially mounted on the auxiliary support part by fitting the inner bore of it with the upper positioning cylinder of the latter. Before these operations, the auxiliary support part was mounted on a multi-axis stage and electrically isolated from the latter, this multi-axis stage and the upper support plate were leveled with reference to the mounting surface of the pneumatic vibration-isolation platform. The precise alignment of the rotor and the stator was achieved by fine-tuning the multi-axis stage to make the initial capacitance of the radial cylindrical electrodes equal. The output capacitance of the T-type CS is read, converted by the signal processing circuit and then transmitted to a host computer through a NI 6259BNC data acquisition card (National Instruments Corporation, Austin, TX, USA). After analyzing and treating by the NI Labview-based program, the change of the output capacitance will be presented.

The multi-axis stage used in this system enables the rotor produce linear translational and tilt motions (to simulate the radial, tilt error motions of the spindle) with the minimum resolutions of 0.01 mm and 1 arc-min respectively; the card NI 6259 BNC can provide the highest 1 MS/s sampling rate and up to 16 channels analog input, which meets the multi-channel and real-time acquisition needs of the experimental data.

### 4.1. Sensor Fabrication

To obtain the required positional accuracy between the surfaces of the rotor which are used for measurement (CS_R_ and the inside annular end face of the F_R_ in Figure 1) and between the annular grooves in the stator housing which are used for mounting the sensing electrode groups, the rotor and stator housing of the T-type CS are both manufactured by a precision CNC machining center, wherein the rotor will undergo a precision grinding procedure so as to achieve a good surface accuracy. The raw material of the rotor is copper which has well electrical conductivity, and the lightweight aluminum alloy is used to manufacture the stator housing.

**Figure 19:**
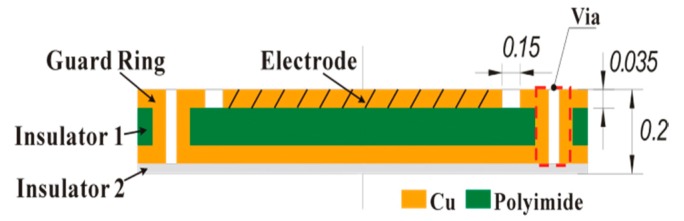
The schematic diagram of the sensing electrode group cross-sectional structure (unit: mm).

**Figure 20:**
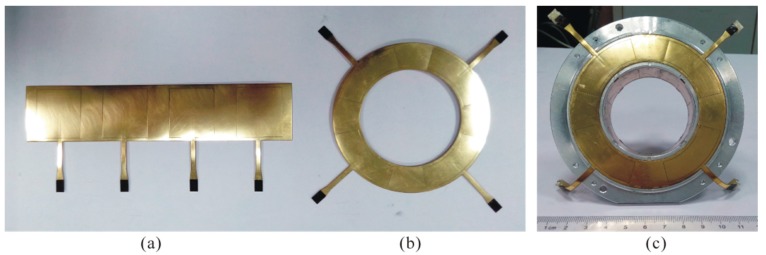
Photograph of the sensing electrode groups and sensor stator: (**a**) Radial sensing electrode group; (**b**) end part sensing electrode group; (**c**) the stator of T-type CS.

As shown in Figure 19, the sensing electrode group of the sensor is fabricated by a four-layer FPCB. The sensing electrode and its peripheral guard ring are fabricated at the top exposed copper foil and a 0.15 mm spacing between them is obtained by the FPCB process, an antioxidant treatment is performed on the copper foil subsequently; the second layer is a polyimide substrate, on the one hand it can achieve electrical insulation between the top-layer electrode and the back protective layer, on the other hand it could provide an auxiliary support to ensure the entire electrode group not only have well deformable, attachable, but also a certain toughness; the third layer is the back protective layer for the sensing electrode, which is connected with the top-layer guard ring through the “vias” (a PCB construction), thereby a nearly closed equipotential protection around the sensing electrode is formed, and meanwhile the external electromagnetic interference is depressed; the bottom overlay is used as the second insulator, for insulating with the stator housing. The design of the entire sensing electrode group was completed by Altium Designer 10 (Altium, San Diego, CA, USA). The resulting sensing electrode groups and the stator after being assembled are shown in Figure 20.

### 4.2. Signal Processing Circuit

**Figure 21:**
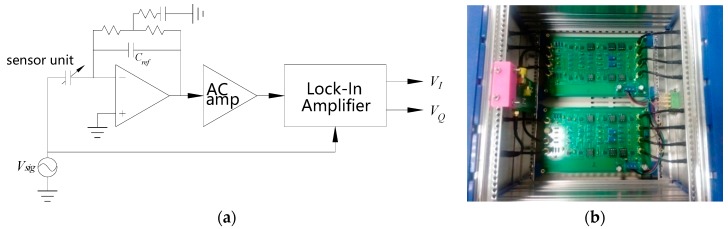
The signal processing circuit of the sensor: (**a**) Block diagram of the principle; (**b**) photograph.

The output signal of the sensor modulated by the excitation electrode (the rotor) position is transmitted to the detection stage of the signal processing circuit through the shielded coaxial cable, as shown in Figure 21. The detection stage is mainly made up of the op amps ADA4077 with a T-configuration resistor network [23], which can effectively pick up the measurement signal and meanwhile avoid the parasitic capacitance generated on the signal transmission path have impact on this stage output result. The output weak signal of the detection stage is amplified by the AC-amp stage configured with a gain of 10 and then is sent to the Lock-In Amplifier [24]. By the Lock-In Amplifier, the enhanced signal is demodulated to the in-phase and quadrature DC components (*V_I_*, *V_Q_*). Then the DC components are converted to digital value and provided to the host computer by the NI 6259BNC. After data processing, the change of the sensor output capacitance will be given.

### 4.3. Measurement Experiments

The experiments were performed based on the above fabricated sensor prototype and circuit, and the simulation results in Section 3 were used as comparative reference during the analysis of the experimental results. Note that C*e in the curve diagrams of the results denotes the experimental value.

#### 4.3.1. Radial Error Motions Measurement

Utilizing the linear translational motion provided by the multi-axis stage, the measurement was carried out to investigate the output capacitance variation of the T-type CS radial cylindrical electrodes, wherein the rotor has radial displacement along y = 0, y = 0.5x and y = x directions respectively. Throughout the measurement process, the variation range of displacement component *dx* is still (−0.2, 0.2) mm and the variation rate is 0.01 mm/step; the amount of *dy* is matched with the *dx* according to the different direction of radial displacement. Affecting by the thickness of the epoxy adhesive layer located between the REG and the stator housing, the actual inside radius of the radial cylindrical electrode in Figure 20c after being assembled is 25.92 mm. According to this measurement result, we modified the radial sensing electrode inside radius of the simulation model in Figure 9 and that of the previously developed CCS in Figure 10 to 25.92 mm. The simulation output of the former and the experimental values of the latter are presented in the following comparative analysis of this section.

**Figure 22:**
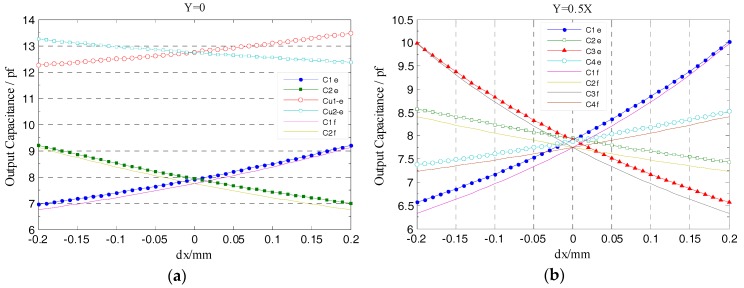
Comparison of experimental and simulation values of the radial cylindrical electrode output capacitance: (**a**) Comparing with the previously developed CCS (2-DOF); (**b**) the comprehensive comparison of the single cylindrical electrode output capacitance. The C*i*e, Cu*i*-e in the curve diagram denote the actual measured values of the T-type CS and the previously developed CCS respectively.

As shown in Figure 22a, the actual measured output capacitance of the T-type CS cylindrical electrode is slightly larger than the simulation values, but is significantly lower compared to that of the previously developed CCS (see Figure 10), which indicates that the T-type CS sensing electrode group which is fabricated by using the FPCB material and meanwhile combining the nearly enclosed equipotential protection scheme could effectively reduce the additional capacitance introduced by fringe effects of the electrode. In the identical error motion direction, such as y = 0, the changing trend of the actual measured output capacitance of the T-type CS cylindrical electrodes are essentially consistent with the simulation values; the variation quantity of those capacitance in the entire displacement range is approximately two times that of the previously developed CCS, and furthermore, this quantity of variation obtained is close to that of the simulation output capacitance.

A relatively comprehensive comparison of the actual measured output capacitance of single electrode as rotor moves along y = 0.5x direction is shown in Figure 22b. Besides keeping basically consistent with the changing trend of the simulation values, the actual measured output capacitance of the two cylindrical electrodes which are located in the staggered quadrants are similarly symmetrically distributed on both sides of the reference line (*dx* = 0), which shows that the cylindrical electrodes among the REG after being assembled have good mutual position accuracy and coaxiality.

**Figure 23:**
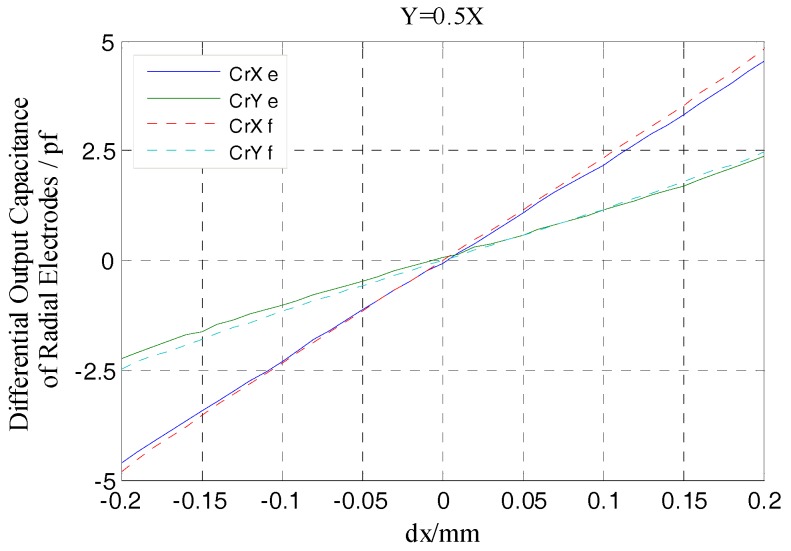
Comparison of experimental and simulation values of the radial differential output capacitance.

According to Equations (9) and (10), the differential output capacitance CrXe, CrYe of the radial electrode group (REG) were obtained by the actual measured output capacitance of single electrodes in Figure 22b and their variations are shown in Figure 23. The result shows that the REG of the T-type CS which is constructed by adopting differential configuration has near-linear output response to the variation of the rotor displacement, i.e., as the measurement proceeds constantly, the capacitance CrXe, CrYe basically exhibit a linear variation. Although due to the developed sensor system has some discrepancies compared to the ideal one, such as in the electrode characteristics or circuit detection performance, the CrXe, CrYe are somewhat smaller with respect to the simulation values CrXf and CrYf, but in any position of the displacement range, the ratio of the CrXe to the CrXf and that of the CrYe to the CrYf both remain stable, which indicates that under different amounts of displacement, a differential output capacitance with good consistency and stability could be obtained utilizing the REG after being assembled and the signal processing circuit constructed.

**Figure 24:**
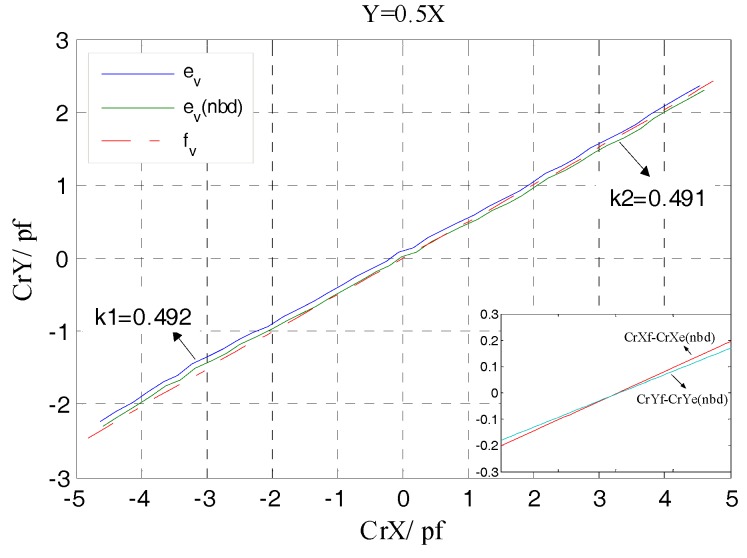
The curve of CrY/CrX in the single radial runout direction.

By taking the differential output capacitance of the REG as dependent variable and variable, respectively, the lines which can also be seen as the trajectory of the rotor are plotted in Figure 24. It can be seen that the curve e_v_ composed by the experimental values CrYe, CrXe has a slight offset with respect to the curve f_v_ of the simulation values. This can be explained by the actual measured output capacitance presented in Figure 22b. Due to factors such as manual operation, assembly fixture precision, there still exists a tiny coaxial deviation *σ* (<0.02 mm) between two cylindrical electrodes and other ones, which makes the actual measured output capacitance of the two electrodes in the 2nd and 4th quadrants appear a little different compared to that of the ones in other quadrants at the origin position (*dx* = 0). This has some effect on the results calculated according to Equations (9) and (10) and leads to the above slight offset of the curve e_v_.

According to the measurement results of the cylindrical electrode geometrical parameters, a compensation for the actual measured output capacitance of the cylindrical electrode based on the origin deviation was performed. The curve e_v_(nbd) which was plotted by the differential output capacitance calculated utilizing the compensation results is presented in Figure 24, and there is no basic offset at the origin. Through linear fitting for the data points that constitute the curve e_v_ and e_v_(nbd), the corresponding slopes k1, k2 are obtained and there is almost no difference between them, which indicates that the tiny coaxial deviation *σ* has no effect on the accurate measurement of the direction angle of the rotor displacement.

**Figure 25:**
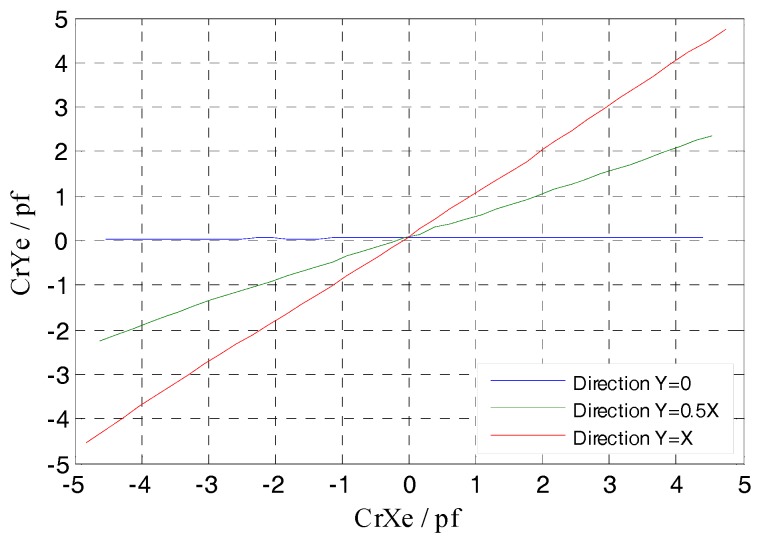
The comprehensive comparison of the curves of CrYe/CrXe in the different radial runout directions.

With respect to the given radial displacement direction (y = 0.5x), the slope k1 of the actual displacement direction obtained from the curve e_v_ is slightly smaller. Due to the fact that the actual displacement that is inputted to the rotor by the manual multi-axis stage has a deviation relative to the theoretical one, a difference is generated between the ratio (CrYf − CrYe(nbd))/(CrXf − CrXe(nbd)) and the ratio of the corresponding difference value between the simulation and theoretical values, which is eventually reflected in the value of slope k1. But from another point of view, the minor difference between the slope of the rotor displacement direction obtained by the actual measurement and the given value indicates that the REG of the T-type CS possesses a resolving capability to the micro direction angle of the radial displacement. For the case when these radial displacements are along different directions, the trajectory line of the corresponding radial displacement which was obtained by the actual measured differential output capacitance CrYe, CrXe is essentially consistent with the given direction, as illustrated in Figure 25.

#### 4.3.2. Tilt Error Motions Measurement

During the mounting process of the T-type CS for the experimental system, the position of the rotor tilt point is adjusted to be consistent with the simulation settings; the initial spacing between the end part fan-shaped electrodes and the axial measuring surface of the rotor is also set to be 1 mm. In this measurement, the *x*-axis of the established fan-shaped electrode coordinate system is parallel to the stator reference edge, and the *y*-axis of it is parallel to a rotation axis of the multi-axis stage. A precision rotation stage is used to adjust the angle between the normal vector of this multi-axis stage rotation axis and the stator reference edge at the horizontal plane, by which the yaw angle *γ* is set to be 15, 30, 45 degrees, respectively, denoted by γ_1_, γ_2_ and γ_3_. At a given yaw angle, the amplitude *ε* is generated in the (−15, 15) arc-min range by fine-tuning the multi-axis stage with a change rate of 1 arc-min/step. The changing situation of the actual measured output capacitance of the end part fan-shaped electrodes were obtained subsequently and investigated as follows.

**Figure 26:**
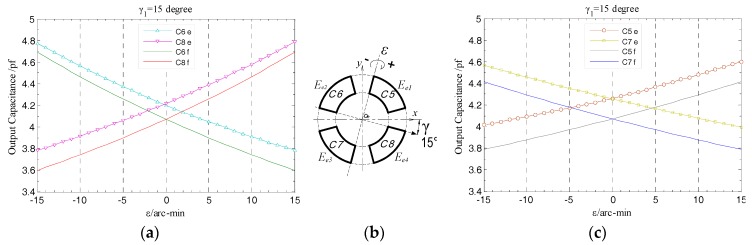
Comparison of experimental and simulation values of the end part fan-shaped electrode output capacitance: (**a**) Comparison between the second and fourth quadrants; (**b**) tilt schematic; (**c**) comparison between the first and third quadrants.

In the case of γ_1_ = 15 degree, as shown in Figure 26, with the change of tilt displacement, the variation tendency of the actual measured output capacitance of the end part fan-shaped electrodes remains basically consistent with the simulation results; besides, the variation magnitude of this capacitance for the fan-shaped electrodes *E*_*e*2_ and *E*_*e*4_ are relatively larger, which is consistent with the analysis given according to Equation (16) in Section 3.2. By means of the integrated and high precision fabrication method, the mutual positional relationship between four-segment fan-shaped electrodes will remain unchanged after the electrode group fabrication is completed, and meanwhile each electrode could attain a well geometric precision. The effectiveness of this method is embodied in the distribution relationship between the actual measured output capacitance of the fan-shaped electrodes which is consistent with the simulation results, i.e., the changing curves of the output capacitance of the two electrodes located in the staggered quadrants are essentially symmetrical relative to the reference position *ε* = 0.

Theoretically, as for the two fan-shaped electrodes located in the staggered quadrants, when the rotor is respectively tilted to the position with equal amplitude but opposite direction, the output capacitance of both of them should be completely equal, but under practical conditions, as illustrated in Figure 26a,c, when the rotor is respectively at the position of *ε* = −15 arc-min and *ε* = 15 arc-min, there is a deviation of approximately 0.02 pf between the corresponding output capacitance of these two electrodes. The reason for generating this result can be given as follows: on the one hand, the flexible property of the FPCB material has a certain impact on the precision of the electrode group contour obtained by laser cutting fabrication, which results in the geometric center of the mounted EPEG not well coinciding with the stator center axis; on the another hand, due to the limitation on the manufacturing precision of the multi-axis stage itself, when fine-tuning the hand wheel of a goniometric stage in it to provide the given tilt motion, an additional slight deflection (arc-sec level) orthogonal to the rotation axis of this tilt is also introduced into the rotor. The former is a systematic error, which could be compensated based on the measured results of the geometric quantities; while the latter is generated by the goniometric stage itself and so has no impact on the measurement accuracy of the T-type CS.

**Figure 27:**
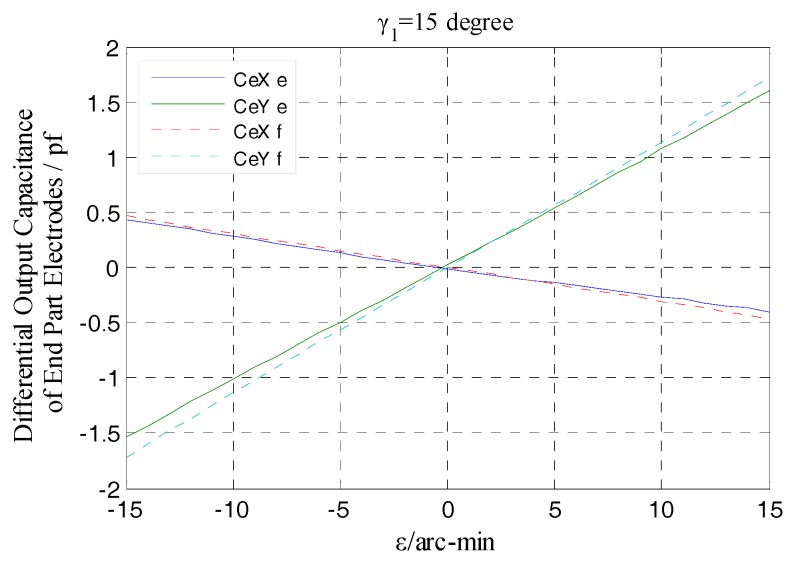
Comparison of experimental and simulation values of the end part differential output capacitance.

The same as the measured output characteristic of the REG, the EPEG of differential configuration also has near-linear output response to the variation of the rotor tilt displacement, as illustrated in Figure 27. Within the whole measurement range, the actual measured differential output capacitance CeXe, CeYe (corresponding to the tilt around *x*-axis, *y*-axis) show a variation tendency that is essentially consistent with the simulation results as the amplitude *ε* of the tilt displacement changes. The EPEG uses the identical materials and fabrication process as the REG and meanwhile was accessed to the same signal processing circuit.

From Figure 27 it can be known that, in any position of the tilt displacement range, the ratio of the differential output capacitance of the EPEG obtained by actual measurement to the simulation value also keeps stable, and this ratio is substantially equal to that of the REG, which is approximately 93%. Thus it can be concluded that, the sensing electrode groups of the developed T-type CS could achieve consistent and stable measurement output for the inputted displacements (linear translation/tilt) and different amount of change in the corresponding type, and the output values are close to the target values; the prototype of the signal processing circuit is effectiveness and has good performance.

**Figure 28:**
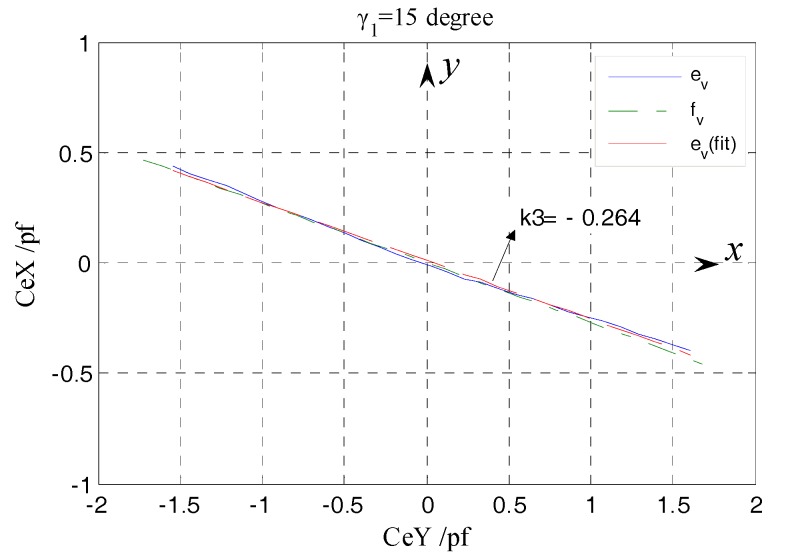
The curve of CeX/CeY as tilting under single yaw angle.

Besides the amplitude *ε* discussed in the above analysis, another element of the tilt error motion needing to be concerned is the yaw angle *γ* shown in Figure 26b. According to Equation (30), the values of the yaw angle are associated with the relative variation between the CeX and CeY, which can be presented using the curve shown in Figure 28. The actual measured curve e_v_ has a slight fluctuation relative to the simulation curve f_v_, due to the influence of random factors on the operation of the stage and the measurement process. By means of the line e_v_(fit) resulting of fitting the data points that constitute the curve e_v_, the yaw direction corresponding to this set of rotor tilt displacement could be shown. Calculating according to the slope k_3_ of the line e_v_(fit), the angle between this yaw direction and the *x*-axis is 14.8° and the relative error of it to the given yaw angle γ_1_ is only 1.3%. The measured yaw direction is substantially in accordance with the preset one.

**Figure 29:**
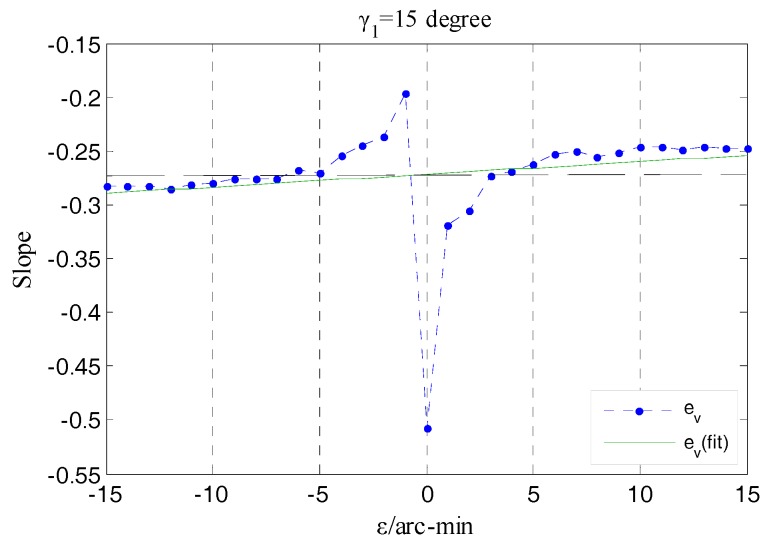
The slope variation for single-step.

Figure 29 shows the variation situation of the values of *k_γ_* (*k_γ_* = tan (*γ*)) that corresponds to the values of yaw angle belonging to the same set of tilt displacements, from a local perspective. In the process where the rotor is moved to the maximum positive tilt position from the maximum negative one, the values of *k_γ_* obtained in each position exhibit a slight decreasing tendency by means of a linear fitting and the corresponding variation is approximately 1.2 × 10^−3^/step (equivalent to the yaw angle changes 3.7 arc-min/step). As mentioned in the comparative analysis of the actual measured output capacitance of single fan-shaped electrodes, when the given tilt displacement is inputted to the rotor, a slight deflection (arc-sec level) orthogonal to the rotation axis of this tilt is also introduced. This results in partial difference between the output capacitance of two electrodes located in the staggered quadrants as the rotor is respectively tilted to the position with equal amplitude but in an opposite direction, and finally is reflected in the relative variation between the CeXe and CeYe, i.e., the values of *k_γ_* corresponding to single-step are diminishing. Because of the correlation between the *k_γ_* and yaw angle *γ*, from another perspective, this experimental result also indicates that the EPEG possesses a measurement capability for the micro yaw angle.

By summarizing each set of CeXe, CeYe values obtained under different given yaw angles γ*_i_* (*i* = 1, 2, 3), the corresponging curves were respectively plotted in Figure 27. Each set of points in this figure is the reference position of one preset yaw direction, while the deflected three lines represent the actual measured yaw directions of the rotor.

**Figure 30:**
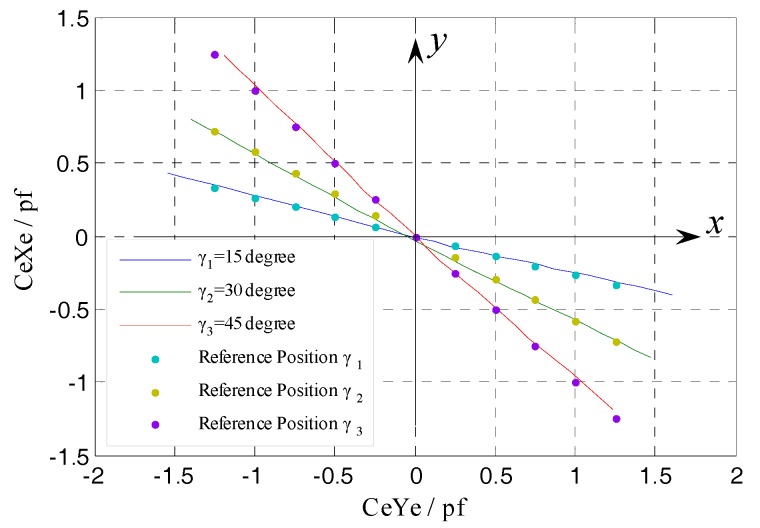
The comprehensive comparison of the curves of CeXe/CeYe under the different yaw angles.

As shown in Figure 30, the actual measured results can accurately reflect the preset yaw directions, which also means that the different amounts of the tilt *ε_x_*, *ε_y_* shown in Figure 14 could be accurately detected. The results indicate that, for the end part fan-shaped electrode which has different shape and measurement purpose (tilt error motion detection) relative to the radial cylindrical electrode, the differential configuration is equally applicable and the expected measurement results can be obtained.

In summary, based on the theoretical modeling and simulation results, the prototype of the T-type CS and of the signal processing circuit were developed. Their feasibility and effectiveness have been verified by actual measurements, while prove the entire measurement system constituted mainly by the foregoing parts possesses the capacity for simultaneously measuring on-axis the 5-DOF error motions of a spindle.

## 5. Conclusions

This paper presents a new method for measuring the 5-DOF error motions of a spindle by using the proposed T-type CS. The proposed T-type CS has an integrated structure which occupies a smaller space and can be easily integrated into a spindle system compared to the existing optical methods; it is only needs to be mounted once for simultaneous measurement of the spindle 5-DOF error motions compared to the method using probe-type capacitive sensors, and is not sensitive to the roundness errors of the reference artifact. The corresponding mathematical models for the measurement of 5-DOF error motions are established; a measurement system prototype constituted mainly by the sensor and signal processing circuit is developed and tested. Details may be summarized as follows:

- The simulation and experimental results indicate that the mathematical models which are established based on the integrated structural model can accurately predict the variation relationship between the inputted displacements (simulating the error motions) and the output capacitance.
- The differential measurement configuration adopted is identically applicable for the sensing electrode groups which have different electrode geometry (curved cylindrical face/planar sector) and different measurement purposes (linear translation/tilt displacement), by which the T-type CS has near-linear output response to the variation of the rotor displacements.
- The feasibility of the proposed fabrication and assembly methods of the sensing electrode group was verified by the measurement results. By using these methods, the additional output capacitance introduced by fringe effects is significantly reduced and the decreasing amplitude is about 90%; the sensing electrode group could be obtained by once machining which makes the electrodes in it have a high geometric precision.
- Based on the constructed signal processing circuit, the actual measured differential output capacitance is up to 93% of the simulation values; furthermore, the T-type CS could achieve consistent and stable measurement output for the inputted displacements.
- The T-type CS only needs to be mounted once times in the measurement process which could effectively avoid the impact of accumulative installation errors on the measurement accuracy.

## Figures and Tables

**Table 1:** Simulation model parameters of T-type CS.

Model Geometry Parameters		Symbol	Value	Unit
Radial cylindrical electrode (Sensing electrode)	Start angle, stop angle (The first quadrant)	*ζ*_1_, *ζ*_2_	π/12, 5π/12	rad mm
	Inside radius	*R*	26	
	Axial width	*w*	30	
	Thickness	*η_r_*	0.1	
End part fan-shaped electrode (Sensing electrode)	Start angle, stop angle (The first quadrant)	*ϕ*_1_, *ϕ*_2_	π/12, 5π/12	rad mm
	Inside radius	*ρ*_1_	30.2	
	Outside radius	*ρ*_2_	42.2	
	Thickness	*η_e_*	0.1	
Rotor (Excitation electrode)	CS_R_ radius	*R_CS_*	25	mm
	Flange radius	*R_F_*	44.2	
Equipotential guard ring	Gap with the sensing electrode	*λ*	0.1	mm
	Thickness	*η_gr_*	0.1	
